# Combining seeded region growing and k-nearest neighbours for the segmentation of routinely acquired spatio-temporal image data

**DOI:** 10.1007/s11548-023-02951-w

**Published:** 2023-06-04

**Authors:** Lukas Zerweck, Stefan Wesarg, Jörn Kohlhammer, Michaela Köhm

**Affiliations:** 1 Fraunhofer Institute for Translational Medicine and Pharmacology ITMP, Frankfurt am Main, 60596 Germany; 2 Fraunhofer Institute for Computer Graphics Research IGD, Darmstadt, 64283 Germany; 3 Fraunhofer Cluster of Excellence Immune-Mediated Diseases CIMD, Frankfurt am Main, 60596 Germany; 4 Division of Rheumatology, Goethe-University Frankfurt, Frankfurt am Main, Germany

**Keywords:** Feature vector based segmentation, Computer vision, Seeded region growing, K-nearest neighbour, Near infrared fluorescence optical imaging

## Abstract

**Purpose:**

The acquisition conditions of medical imaging are often precisely defined, leading to a high homogeneity among different data sets. Nonetheless, outliers or artefacts still appear and need to be reliably detected to ensure a reliable diagnosis. Thus, the algorithms need to handle small sample sizes especially, when working with domain specific imaging modalities.

**Methods:**

In this work, we suggest a pipeline for the detection and segmentation of light pollution in near-infrared fluorescence optical imaging (NIR-FOI), based on a small sample size. NIR-FOI produces spatio-temporal data with two spatial and one temporal dimension. To calculate a two-dimensional light pollution map for the entire image stack, we combine *region growing* and *k-nearest neighbours* (kNN), which classifies pixels into fore- and background by its entire temporal component. Thus, decision-making on reduced data is omitted.

**Results:**

We achieved a $F_1$ score of 0.99 for classifying a data set as light polluted or pollution-free. Additionally, we reached a total $F_1$ score of 0.90 for detecting regions of interest within the polluted data sets. Finally, an average Dice’s coefficient measuring the segmentation performance over all polluted data sets of 0.80 was accomplished.

**Conclusions:**

A Dice’s coefficient of 0.80 for the area segmentation does not seem perfect. However, there are two main factors, besides true prediction errors, lowering the score: Segmentation mistakes on small areas lead to a rapid decrease in the score and labelling errors due to complex data. However, in combination with the light-polluted data set and pollution area detection, these results can be considered successful and play a key role in our general goal: Exploiting NIR-FOI for the early detection of arthritis within hand joints.

## Introduction

Specialized or domain specific imaging modalities in medicine often suffer from a lack of data, let alone annotated data. For these imaging modalities, computer vision tasks, including classification, segmentation or other data analytics need to handle small sample sizes, often restricting the use of highly customized neural networks, the state of the art in computer based decision-making.

In this work, we present a segmentation algorithm for spatio-temporal (two spatial dimensions *x* and *y* and one temporal dimension *t*) image stacks, for the use case of identifying light pollution in near infrared fluorescence optical imaging (NIR-FOI). The goal of the present work is to identify a two-dimensional map per data set, defining all regions for the whole image stack in which light pollution occurs.

**Fig. 1:**
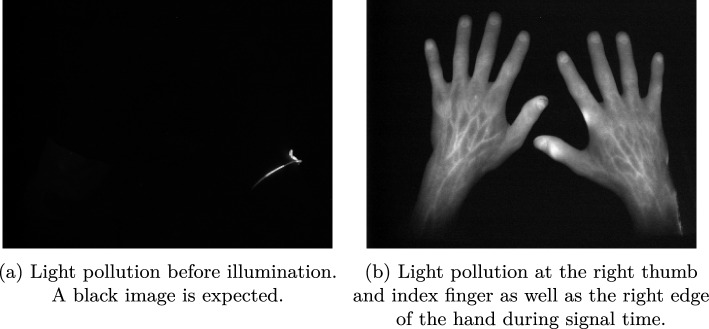
Two samples of light-polluted data sets

Our general research goal is to exploit NIR-FOI to detect arthritis in all joints located in the hands. Based on the distribution process of a colour agent, predictions about the health status (inflamed vs. not inflamed) of an investigated joint are made. Thus, the data analysis of the temporal component focuses on the illumination process rather than object motion. Therefore, the correct localization of ambient light is of utmost importance to separate between colour agent-based and ambient light-based illumination.

The development of a robust segmentation algorithm faces several challenges. In our case, the number of data sets with light pollution is very small (18), and the temporal components among different data sets and the phenotypes of different light pollutions are very heterogeneous. Due to the heterogeneity, an approach not only adapting to the data set but also to each light pollution area is necessary to correctly segment the areas of interest. Additionally, in many cases, the core of the polluted area is well visible and easier to detect than the pixels towards the edges. Thus, a data reduction, based on a pre-set of parameters, along the temporal component followed by a segmentation step often leads to unsatisfying results, either over-segmenting or leaving out less dominant areas towards the edges of the polluted areas. The low performance of this two-step approach is caused by the mentioned parameter definitions, which might lead to satisfying results in some cases however, fails for other cases not meeting the assumptions baked into the parameter definitions. The poor segmentation of this two-step approach was observed independently of the reduction method, for example *feature value extraction*, *PCR analysis*, *Fourier analysis* etc. Therefore, a segmentation pipeline based on none reduced temporal data in the *t* dimension is necessary. Two samples of light-polluted data sets are shown in Fig. 1.

In order to address all these conditions, we developed a segmentation pipeline based on two well-known algorithms: *seeded region growing* [1] and *k-nearest neighbours* (kNN) [2]. The general idea is to use the seeded region growing algorithm to test whether the next pixel belong to the current area. However, instead of pre-defining an inclusion criterion, the decision is made by a pre-trained machine-learning model (kNN). Our approach combines the benefits of a classical segmentation algorithm, which does not require training data, and a lightweight machine-learning algorithm highly adapted to its’ training data. This combination leads to a minimum of pre-defined parameters, is highly adaptive to each polluted area, and does not require multiple data sets for model training.

The usage and dimensions of the NIR-FOI data lead to a unique set of requirements for the data analysis, including the image segmentation for ambient light-polluted areas. Thus, to aid in comprehensibility of the *Related work* chapter, especially the comparison of the work at hand to previous work, the *Data* chapter is located before the *Related work* chapter.

## Data

NIR-FOI is an imaging modality, in which the colour agent indocyanine green (ICG) is administered intravenously. During the following six minutes, 360 images of the colour agent’s distribution are taken leading to the three dimensions mentioned in the “Introduction” section, in which the spatial dimensions *x* and *y* refer to each slice’s pixels and *t* to the consecutive image acquisition.

**Fig. 2:**
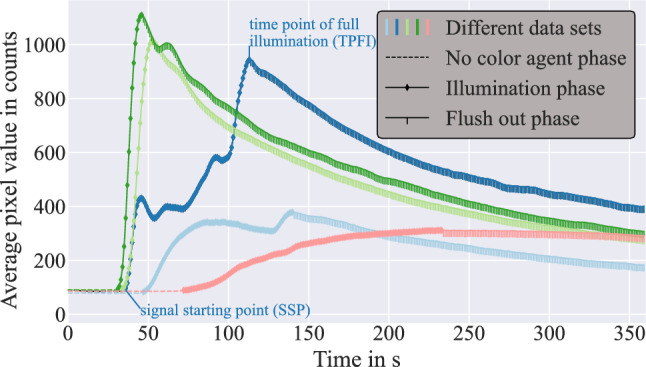
Five exemplary timelines ([3] adjusted design)

Each data set consists of three periods separated by two distinguishable points in time: Prior signal, signal starting point (SSP), signal increasing time, time point of full illumination (TPFI) and signal decreasing time. Figure  2 displays the average count value (measured pixel value) of each slice, visualizing these phases and points in time. Phase one refers to the period prior to the ICG reaching the hands (No colour agent (NoCA) phase). Then, the ICG reaches the hands (SSP) and the second phase, in which the colour agent distributes throughout the blood vessels, starts (illumination phase). In the third phase, after reaching the TPFI, the ICG is flushed out of the microcirculation in the hands, which leads to a decrease in pixel count (flush-out phase). The starting points and length of each phase vary among different data sets. This definition of phases and distinguishable points in time was already introduced in [3].

**Fig. 3:**
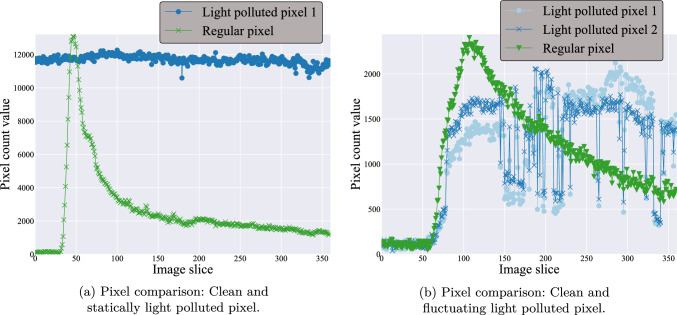
Examples of data affected by light pollution

The used data for this work were acquired during four independent studies, each of which fulfilled Good Clinical Practice Guidelines in accordance with the Declaration of Helsinki and were approved by the ethics committee of the University Hospital Frankfurt a. Main, Germany (No.: 127/13, No.: 128/13, No.: 433/18, No.: 454/17). All participants provided signed informed consent for inclusion in the studies and agreed to the usage of their data for research purposes. All participants were fully capable of giving informed consent for participation in a study.

### Light pollution

Generally, the image acquisition room should be darkened as much as possible. However, in some cases, doors or windows are not covered or the room lights are not switched off, leading to ambient light being measured by the experimental setup. Therefore, the light pollution is affected by the surrounding conditions, which can lead to diverse phenotypes of light pollution. It can be all gradation from a statically increased area to a highly fluctuating signal. Figure 3a shows the comparison between a statically affected pixel and an unpolluted pixel. Figure 3b displays two pixels of the same light-polluted area, in which during the acquisition period the signal starts to fluctuate. Furthermore, the timeline of an unpolluted pixel is added.

## Related work

As mentioned in the “Introduction” Section, the goal of the present work is a two-dimensional map per data set, localizing all pixels, which have been polluted at some point during the 360 images. This correlates with a dimension reduction from a three-dimensional data set (*x*, *y* and *t*) to a two-dimensional (*x* and *y*) segmentation map.

In many cases, a segmentation of all dimensions is required. For a three-dimensional volume (three spatial dimensions), a three-dimensional segmentation defines the three-dimensional shape of an object. A survey about that topic is given in [4]. For spatio-temporal data, a slice-wise segmentation localizes moving or distorted objects at every given point in time, again a segmentation in all three dimensions. A survey about spatio-temporal data in today’s medicine is given in [5]. However, in this work we interpret the time series per pixel as feature vector, which is closer to texture-based segmentation approaches. Nonetheless, these approaches apply wavelet-filter through convoluting filters with images [6]. Due to the high heterogeneity among different data sets as well as different polluted areas, global filtering did not lead to satisfying results. Thus, the main difference to previous work is the individually trained kNN classifiers for each polluted area to achieve maximal adaptation.

To our knowledge, there is no other research using the result of a machine-learning classifier as inclusion criteria in a seeded region growing algorithm. There have been approaches to increasing the performance of region growing by combining it with other techniques such as edge detection [7], graph based methods [8], advanced thresholding [9, 10], increasing the region growing’s adaptiveness [11] and others. However, none of these methods builds their segmentation decision on a multidimensional input vector.

The only work we found, suggesting a higher dimension decision-making for a lower dimension segmentation result is [12], in which a multistep process is suggested for the segmentation and merging of related areas. In the work at hand, a multistep process, extracting a feature map, which is then used for the segmentation, leads to unsatisfying results.

To our knowledge, there is no other research investigating the ambient light pollution in NIR-FOI and its possible influence on the medical decision-making. The presented work is a first into this direction.

## Method

All described methods were implemented using Python, with extensively using the following modules: NumPy [13], OpenCV [14] and scikit-learn [15].

The main focus of this work is the combination of the seeded region growing algorithm with the kNN classifier. However, as in most machine based decision-making, the definition and preparation of training data and parameters through empirical testing or classic data analysis steps (hyperparameter tuning) is a major part of the process. In the work at hand, “Determining ambient light pollution” and “Defining area seeds and trainings data” describe these pre-processing steps. The complete segmentation pipeline contains the following three main steps:

1. Determine whether the current data set is polluted by ambient light
2. If ambient light was detected, defining the seeds of and training data for the seeded region growing and kNN classifier
3. Perform the segmentation for each detected region

If a pixel is classified as light polluted at any given time point, it is removed from any calculation for the entire image stack.

### Determining ambient light pollution

As mentioned in “Data”, the data is composed of three phases (see Fig. 2). Since the requirements and conditions differ for these phases, the ambient light detection is performed individually for each phase. However, the signal characteristic of the illumination phase is a rapidly changing signal, which can be misinterpreted as ambient light pollution. To avoid misclassification, no ambient light detection is carried out in this phase. Furthermore, in none of the data sets, a pollution area solely appearing in phase 2 has been observed by a qualified human observer.

Even though the high heterogeneity among different data sets encourages the usage of relative thresholds to perform adapted decision-making, the classification into ambient light-polluted and clean data sets require an absolute statement valid for all data sets. For the two phases, individual transformations and different absolute conditions (numerical thresholds) are defined through intensive testing and fine-tuning.

The results, after applying transformations and general thresholding, are binary light pollution maps defining foreground and background (Figs. 5b and 8). However, these are not precise segmentation masks, but rather identify the core regions of the light pollution areas. These are used for the seed and training data definition in “Defining area seeds and trainings data”.

**Fig. 4:**
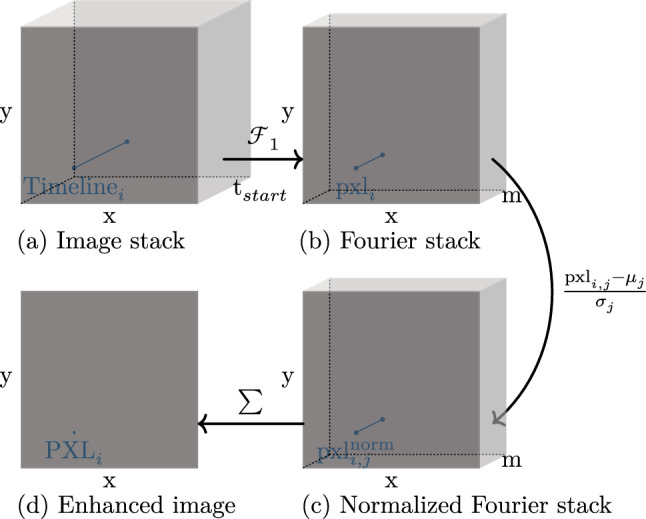
Calculation pipeline to extract an image emphasising light-polluted areas. This graphic was created with the tool TikZ [16]

#### NoCA phase

The temporal component of the no colour agent phase is usually 15 to 150 images long, in which only black noisy images are expected. However, due to the high heterogeneity, defining hard threshold values, classifying too bright pixels as ambient light, leads to misclassification. Therefore, multiple steps are carried out to incorporate these diverse conditions.

**Fig. 5:**
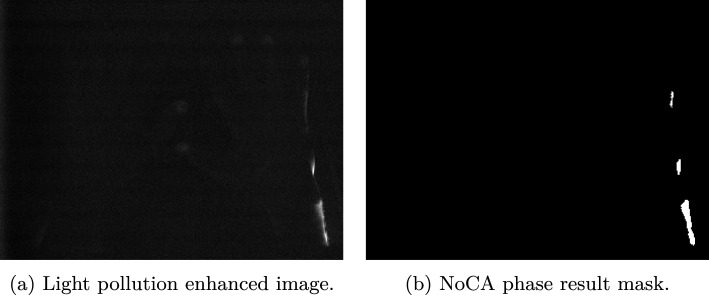
Results of the flush-out phase

Firstly, an image, which emphasises light-polluted pixels, is calculated. The whole process is visualized in Fig. 4. In a first step, the timelines of all $x\cdot y = n$ pixels are transformed into the frequency space using the one-dimensional Fourier transformation. Since this transformation yields complex numbers, the absolute values are calculated to obtain a real value result. Additionally, only half of the Fourier values per pixel are kept, due to the mirror characteristic of the results of a Fourier transformation (Fig. 4a to b). Then, the z-score for each Fourier component among all *n* pixels is calculated (Fig. 4b to c). The mathematical foundation of the normalization process is given in Eq. 1, in which $\text {pxl}_{i,j}$ refers to the pixel value in the Fourier stack with image index *i* and component index *j*. $p_{i,j}$ describes the likelihood of the appearance of $\text {pxl}_{i,j}$ ($p_{i,j} = \frac{\#\,\,{\text { of appearance pxl}}_{i, j}}{n}$).

1 $$\begin{aligned} \mu _j&= \frac{1}{n}\displaystyle \sum _{i=0}^{n-1} \text {pxl}_{i, j}~,~j \in \{0,1,...,m-1\}\nonumber \\ \sigma ^2_j&= \displaystyle \sum _{i=0}^{n-1} \big (\text {pxl}_{i, j} - \mu _j\big ) \cdot p_{i,j}~,~j \in \{0,1,...,m-1\}\nonumber \\ \text {pxl}^{\text {norm}}_{i,j}&= \frac{\text {pxl}_{i,j} - \mu _j}{\sigma _j} ~,\nonumber \\&\quad \quad \quad ~ i \in \{0,1,...,n-1\}~,~~j \in \{0,1,...,m-1\} \end{aligned}$$

After each value in the Fourier stack is normalized, the sum along the component axis of every pixel is calculated (Fig. 4c to d).

2 $$\begin{aligned} \text {PXL}_i = \displaystyle \sum _{j=0}^{m-1} \text {pxl}^{\text {norm}}_{i,j} ~,~ i \in \{0,1,...,n-1\} \end{aligned}$$

*m* refers to the number of Fourier components and $\hbox {PXL}_i$ to the pixel value of the calculated image (Fig. 4d). One example of such an image is visualized in Fig. 5a.

With this component-wise normalization, two things are achieved. Firstly, each pixel lies in the same numerical range, independent of the original amplitude of the image stack. Secondly, Fourier components with lower original amplitudes, usually high frequencies, increase relatively in comparison with components with higher amplitudes, usually low frequencies. Since high frequencies represent detailed image information, in the data at hand bright stack pixels, the areas of interest, are emphasised. These two properties enable a general thresholding to classify each pixel into ambient light-polluted or clean data. A result of the NoCA phase is visualized in Fig. 5b.

#### Flush-out phase

**Fig. 6:**
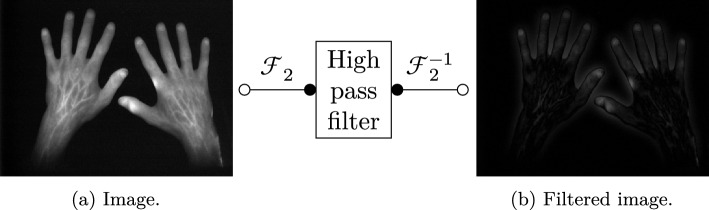
High-pass filter pipeline for one example image

Extracting a light pollution map for this phase has different requirements in comparison with the NoCA phase. The main difference is the existing signal from the ICG and thus, the necessity to separate signal and ambient light pollution. As mentioned in the “Method” section, every pixel, which was classified as light polluted during the NoCA phase, is discarded from the entire image stack. Therefore, in this phase the focus lies on rapid unexpected signal changes and not on statically increased signals, since these will have been detected during the NoCA phase. The flush-out phase spans from TPFI (see Fig. 2) to the end of the data set.

**Fig. 7:**
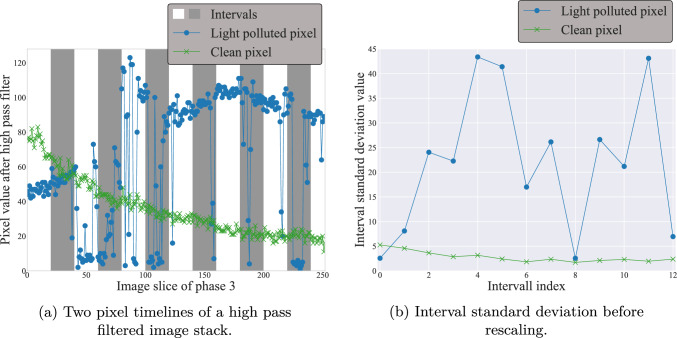
Visualization of the interval-wise standard deviation calculation

Each image in the defined flush-out phase image stack is filtered by a Gaussian high-pass filter, which is performed by transforming each image in the spatial frequency space via a two-dimensional discrete Fourier transformation. This high-pass filter emphasises details in the images, for example light-polluted areas, and decreases general image information such as low-resolution hand parts (an example is given in Fig. 6).

To achieve a two-dimensional light pollution map, the temporal component has to be removed. Therefore, the standard deviation along the temporal component for each pixel is calculated. The standard deviation increases numerically, when the values are distributed over a wider range. However, as seen in Fig. 2 the data sets tend to decrease in signal amplitude after the TPFI has been passed. This leads to a decrease in impact for numerically smaller values when calculating the standard deviation. To account for this phenomenon, the standard deviation along the temporal component of the high-pass-filtered image stack is calculated consecutively for intervals of the image stack. These intervals are shown in Fig. 7a. After the standard deviation image is calculated for one interval, it is normed into $\big \{x \in {\mathbb {R}}~\vert ~0\le x \le 1 \big \}$ over all pixels.

**Fig. 8:**
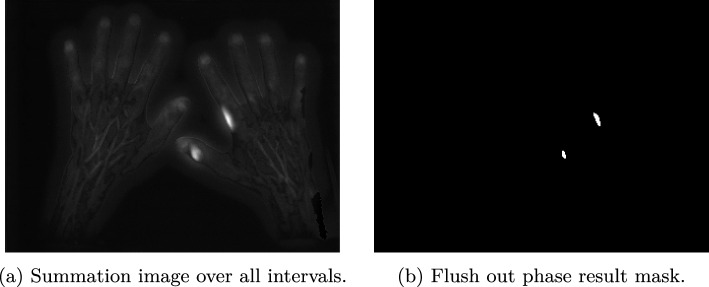
Results of the flush-out phase

Repeating this process for all intervals flattens the decreasing signal, puts values across all pixels into relation, and transforms all values into the same numerical scale (an example is given in Fig. 7a). After all normed interval standard deviation images are calculated, the sum among all these images is calculated and the image rescaled into the range $\big \{x \in ~{\mathbb {N}}\vert ~0\le x \le 255 \big \}$ (unsigned integer with bit size 8). A summation image is shown in Fig. 8a. The data from different data sets is comparable after this process and general thresholds can be applied. A final result of the flush-out phase is visualized in Fig. 8b.

### Defining area seeds and trainings data

Preparing the data and defining the seeds are done separately for the result masks of the NoCA and flush-out phase. However, the process is identical and therefore only described once.

**Fig. 9:**
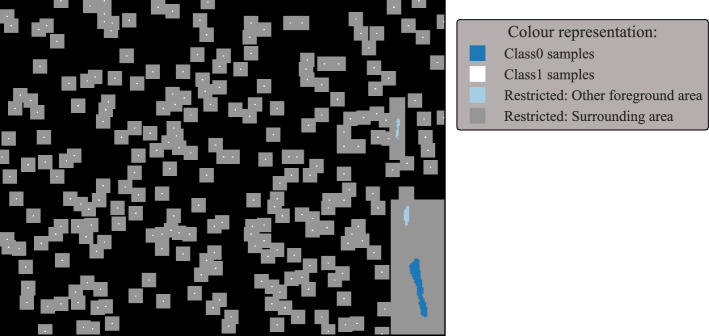
Visualization of the kNN trainings data

As mentioned before, the suggested segmentation method combines seeded region growing and a kNN classifier. Thus, the seeds and the training data need to be defined.

#### Seed definition

The seeds’ definition is straight forward, after receiving the result mask (e.g. Figs. 5b or 8b). For each detected area, the seed is defined by calculating the average pixel indices of both dimensions *x* and *y*, including all pixels in the area.

3 $$\begin{aligned} \text {seed}_{j,d}= & {} \bigg \lfloor \frac{1}{n_j}\sum _{i=0}^{n_j}\text {pnt}_{j,i,d} \bigg \rfloor \nonumber \\{} & {} \quad \forall ~j \in {\mathbb {N}}:0\le j < Q~,~d \in \{x,y\} \end{aligned}$$

*Q* describes the number of detected foreground areas, $\hbox {pnt}_{j,i}$ describes the *i*th point in the *j*th foreground area and $d \in \{x,y\}$ symbolize the respective dimension.

#### Defining the trainings data

It is assumed that pixels belonging to the same foreground area have a similar light pollution (e.g. the fluctuation is similar). However, other foreground areas might show a different temporal behaviour. Thus, similar to the seeds’ definition, the kNN training data needs to be defined individually for each foreground mask. Additionally, as mentioned in “Introduction”, the inclusion of a pixel during the region growing process should be based on the entire pixel’s timeline of the current phase. Therefore, the timelines of all pixels labelled as foreground of one individual area are defined as the training samples for one class. This is Class1 and describes the foreground.

The second class Class0 describes the background. Pixels and their timelines are randomly picked from the rest of the image and labelled as Class0. However, some restrictions apply to the randomly picked indices. Firstly, all defined foreground areas and a larger surrounding region are prohibited to be picked for background pixels. Additionally, each picked pixel gets a restricted area assigned, from which no further pixel can be selected. This avoids too closely located pixels to be picked as training data, to get a more diverse Class0 training data set. The number of Class0 samples is set to the number of Class1 samples to avoid class imbalance. An example is shown in Fig. 9.

### Calculating the light-polluted areas

After the training data for one region of interest is defined, it is used to train the kNN classifier. Starting from the calculated seed, the eight surrounding pixels are classified and added to the region of interest, should the classifier predict the currently tested pixel as Class1. Otherwise, it is not included. Pixels included into the region of interested will be used as the next core pixels, from which yet untested pixels will be tested. This process is repeated until no further core pixel is included into the foreground (region growing). Especially in the beginning, many pixels used as Class1 training data are tested. However, if reaching a pixel, which was used as Class1 trainings pixel, the timeline is not classified but simply accepted as a Class1 pixel. In comparison, pixels of Class0 are not automatically classified as Class0. In some cases, the region of interest grows beyond the predefined restricted area around the core region and thus, Class0 pixels can be reached and possibly classified as Class1.

The hyperparameter *k* of the kNN classifier, defining how many votes are included into the majority vote, is set to 3.

## Results

In order to evaluate the performance of the segmentation pipeline, three different metrics are calculated: Dice’s coefficient $\chi$ [17] for the pixel wise light pollution area segmentation per data set, $F_1$ score $\psi$ for the general area detection and the $F_1$ score $\tau$ classifying data sets into light polluted and free of pollution. Dice’s coefficient is a measure to estimate the similarity between two sets. For two binary data sets, Dice’s coefficient and $F_1$ are the same and defined as:

4 $$\begin{aligned} F_1 = \frac{2\cdot \textrm{TP}}{2\textrm{TP}+\textrm{FP}+\textrm{FN}} \end{aligned}$$

wherein the number of true positive (TP), false positive (FP) and false negative (FN) of the respective classification are used. In case of $\chi$, the evaluation of the segmentation performance, TP describes the pixels correctly segmented, FP pixels labelled as background but segmented as foreground and FN pixels labelled as foreground but segmented as background. For $\psi$, the evaluation of the area detection, a region is defined as detected, if at least one pixel of a labelled area matches a segmented pixel.

As mentioned in the “Data” section, 18 samples contain light pollution. Furthermore, the pipeline was tested on 57 additional data sets not containing any light pollution. Out of these 57 data sets, 56 are identified as pollution-free and one as polluted. All 18 polluted data sets were correctly detected. This leads to:

5 $$\begin{aligned} \tau = \frac{2\cdot \overbrace{(18 + 56)}^{\textrm{TP}=74}}{2\cdot \underbrace{74}_\textrm{TP} + \underbrace{1}_\textrm{FP} + \underbrace{0}_\textrm{FN}} = 0.99. \end{aligned}$$

**Fig. 10:**
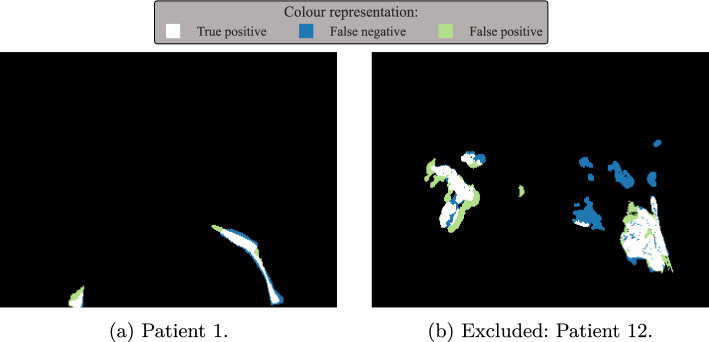
Segmentation results

The results for $\chi$ and $\psi$ are summarized in Table 1. An average Dice’s coefficient of $\overline{\chi }=0.8$ and a total $F_1$ score of $\psi _\textrm{tot}=0.899$ for area detection were achieved. The segmentation result of patient 1 is shown as an example in Fig. 10a. Furthermore, to define a correct segmentation label for patient number 12 seems impossible, since the patient’s hands seem to be covered in a fluorescent substance. A decision, whether a pixel belongs to the fore- or background, is nearly impossible. Thus, it is excluded from the calculations for $\overline{\chi }$ and $\psi _{tot}$. However, labelling this sample as light polluted is possible, and therefore it is included into the $\tau$ calculation (see Eq. 5). The segmentation result for this excluded sample is visualized in Fig. 10b.

**Table 1:** Summarized results of the segmentation pipeline

Patient	Dice’s $\chi$	Area evaluation			
		TP	FN	FP	Dice’s $\psi$
1	0.71	2	0	0	1.00
2	0.82	2	0	2	0.67
3	0.62	1	0	0	1.00
4	0.67	1	0	0	1.00
5	0.73	3	1	0	0.86
6	0.67	5	2	0	0.83
7	0.82	1	0	0	1.00
8	0.82	2	0	0	1.00
9	0.96	1	0	0	1.00
10	0.71	4	0	2	0.80
11	0.92	1	0	0	1.00
12	0.65	4	6	1	0.53
13	0.93	1	0	0	1.00
14	0.91	1	0	0	1.00
15	0.89	1	0	0	1.00
16	0.90	1	0	0	1.00
17	0.82	1	0	0	1.00
18	0.73	3	0	0	1.00
	Ø	$\sum$	$\sum$	$\sum$	
	**0**.**80**	**31**	**3**	**4**	**0**.**90**

## Discussion and conclusion

In this work, we present a segmentation algorithm for spatio-temporal data, with high heterogeneity among different but limited amount of samples. Our goal is to incorporate the properties of the temporal component into the decision-making, whether a pixel belongs to a specific foreground area.

With the presented pipeline, we achieved a $F_1$ score of $\tau =0.99$ in detecting light-polluted or pollution-free data sets. Over all polluted data sets, a $F_1$ score of $\psi _{tot}=0.90$ in detecting areas of interest and an average Dice’s coefficient of $\overline{\chi } = 0.80$ with $\chi _\textrm{min}=0.62$ for the segmentation were achieved. In particular, the detection of light-polluted data sets ($\tau$) and the corresponding areas ($\psi _{tot}$) can be considered well working. The area segmentation ($\chi$) also achieves in most cases satisfying results. Besides true prediction errors, low values have two leading causes. Firstly, some areas are small in size, and thus small differences between label and prediction yield relatively low values, a property of the evaluation measure. Secondly, in order to evaluate $\chi$ a ground truth mask based on multiple images (temporal component) needs to be defined. This complex task can lead to uncertain labels and therefore lower performance. Nonetheless, the achieved results are considered well working for our application, since the main goal of this research is the assessment of whether automatically investigate the current data set or not. The combination of $\tau =0.99$, $\psi =0.90$ and $\overline{\chi }=0.80$ enables to automatically assess whether a data set contains ambient light pollution and if so, the effects the pollution has on evaluating critical hand regions.

The two major challenges of this pipeline are the definition of seeds and training data. This pre-processing step is highly dependent on the use case and might not be transferable to other research questions. Nevertheless, the main idea of combining region growing and machine-learning can be easily expanded to a diverse set of topics and applications (e.g. other types of angiography with a temporal component). Whenever a segmentation should be based on higher dimensional data, this approach can be applied. Furthermore, this method is highly adaptive to the data and can be, if required, expanded by more comprehensive machine-learning approaches such as neural networks.

## References

[1] Adams R, Bischof L (1994). Seeded region growing. IEEE Trans Pattern Anal Mach Intell.

[2] Cover T, Hart P (1967). Nearest neighbor pattern classification. IEEE Trans Inf Theory.

[3] Zerweck L, Wesarg S, Kohlhammer J, Köhm M (2023) Machine Learning Based Approach for Motion Detection and Estimation in Routinely Acquired Low Resolution Near Infrared Fluorescence Optical Imaging. Springer Cham 13746. Accepted for publication

[4] He Y, Yu H, Liu X, Yang Z, Sun W, Wang Y, Fu Q, Zou Y, Mian A (2021) Deep Learning based 3D Segmentation: A Survey. CoRR. arxiv:2103.05423

[5] Hernandez KAL, Rienmüller T, Baumgartner D, Baumgartner C (2021). Deep learning in spatiotemporal cardiac imaging: a review of methodologies and clinical usability. Comput Biol Med.

[6] Arivazhagan S, Ganesan L (2003). Texture segmentation using wavelet transform. Pattern Recogn Lett.

[7] Zhou Y, Starkey J, Mansinha L (2004). Segmentation of petrographic images by integrating edge detection and region growing. Comput Geosci.

[8] Oghli MG, Mohammadzadeh M, Mohammadzadeh V, Kadivar S, Zadeh AM (2017). Left ventricle segmentation using a combination of region growing and graph based method. Iran J Radiol In press (In press).

[9] Stewart RD, Fermin I, Opper M (2002). Region growing with pulse-coupled neural networks: an alternative to seeded region growing. IEEE Trans Neural Netw.

[10] Lu Y, Miao J, Duan L, Qiao Y, Jia R (2008). A new approach to image segmentation based on simplified region growing PCNN. Appl Math Comput.

[11] Pohle R, Toennies KD (2001) Segmentation of medical images using adaptive region growing. In: Sonka, M. Hanson, K. M. (ed) Medical Imaging 2001: Image Processing, 4322: 1337–1346. SPIE. 10.1117/12.431013

[12] Chan DY (2005). Image segmentation with fast wavelet-based color segmenting and directional region growing. IEICE Trans Inf Syst.

[13] Harris CR, Jarrod Millman K, van der Walt SJ, Gommers R, Virtanen P, Cournapeau D, Wieser E, Taylor J, Berg S, Smith NJ, Kern R, Picus M, Hoyer S, van Kerkwijk MH, Brett M, Haldane A, Fernández del Río J, Wiebe M, Peterson P, Gérard-Marchant P, Sheppard K, Reddy T, Weckesser W, Abbasi H, Gohlke C, Oliphant TE (2020) Array programming with NumPy. Nature 585(7825):357–362. 10.1038/s41586-020-2649-2 10.1038/s41586-020-2649-2 PMC7759461 32939066

[14] Bradski G (2000) The OpenCV library. Dr. Dobb’s J Softw Tools

[15] Pedregosa F, Varoquaux G, Gramfort A, Michel V, Thirion B, Grisel O, Blondel M, Prettenhofer P, Weiss R, Dubourg V, Vanderplas J, Passos A, Cournapeau D, Brucher M, Perrot M, Duchesnay E (2011). Scikit-learn: machine learning in python. J Mach Learn Res.

[16] Tantau T The TikZ and PGF Packages. http://sourceforge.net/projects/pgf/

[17] Dice LR (1945). Measures of the amount of ecologic association between species. Ecology.

